# The Early Detection of Osteoporosis in a Cohort of Healthcare Workers: Is There Room for a Screening Program?

**DOI:** 10.3390/ijerph18031368

**Published:** 2021-02-02

**Authors:** Carmela Rinaldi, Sara Bortoluzzi, Chiara Airoldi, Fabrizio Leigheb, Daniele Nicolini, Sophia Russotto, Kris Vanhaecht, Massimiliano Panella

**Affiliations:** 1 Department of Translational Medicine, University of Eastern Piedmont (UPO), 28100 Novara, Italy; sara.bortoluzzi@uniupo.it (S.B.); chiara.airoldi@uniupo.it (C.A.); fabrizio.leigheb@med.uniupo.it (F.L.); daniele.nicolini@uniupo.it (D.N.); massimiliano.panella@med.uniupo.it (M.P.); 2 University Hospital “Maggiore della Carità”, 28100 Novara, Italy; 3 School of Medicine, University of Eastern Piedmont (UPO), 28100 Novara, Italy; sophiarussotto@yahoo.com; 4 KU Leuven Institute for Healthcare Policy, 3000 Leuven, Belgium; kris.vanhaecht@kuleuven.be

**Keywords:** osteoporosis, screening, prevention, public health, worker health, workplace

## Abstract

Workforce aging is becoming a significant public health problem due to the resulting emergence of age-related diseases, such as osteoporosis. The prevention and early detection of osteoporosis is important to avoid bone fractures and their socio-economic burden. The aim of this study is to evaluate the sustainability of a screening workplace program able to detect workers with osteoporosis. The screening process included a questionnaire-based risk assessment of 1050 healthcare workers followed by measurement of the bone mass density (BMD) with a pulse-echo ultrasound (PEUS) at the proximal tibia in the at-risk subjects. Workers with a BMD value ≤ 0.783 g/cm² were referred to a specialist visit ensuring a diagnosis and the consequent prescriptions. Any possible association between the outcome variable BMD ≤ 0.783 g/cm² and the risk factors was evaluated. The costs were calculated with a full costing method. We identified 60 pathological subjects. We observed increased risks for women, older ages, and menopause (*p* < 0.01). The yearly cost of our screening program estimated for this study was 8242 euros, and, considering the fragility bone fracture costs, we hypothesize a considerable economic savings, with a possible positive benefits/cost ratio of 2.07. We can say that the margin between the investment and results leads to a preference for this type of screening program. Osteoporosis is an occupational health problem, and a workplace screening program could be a cost-effective intervention.

## 1. Introduction

Workforce aging is becoming a significant occupational health issue in many countries [1]. As a consequence, age-related conditions and diseases are quickly emerging in the workplace, with a detrimental impact on workers’ physical functions and productivity. Osteoporosis is a good example of these conditions. This disease affects more than 200 million people worldwide, and the osteoporosis burden is likely to increase substantially in the near future with negative outcomes in terms of bone fractures, mortality, quality of life reductions, chronic pain, autonomy loss, and social costs [2,3].

The risk factors involved in osteoporosis are divided into major modifiable and major non-modifiable risk factors. Major modifiable risk factors include inadequate nutritional absorption, a lack of physical activity or fall risk, weight loss, cigarette smoking, and alcohol consumption. Major non-modifiable risk factors include a history of falls, older age, gender, white ethnic background, prior fracture, and reproductive factors (family history of osteoporosis). Then, we have secondary causes of osteoporosis: chronic use of certain medications (prolonged corticosteroid use and so on), hypogonadism, hyperparathyroidism, chronic liver disease, inflammatory diseases (rheumatoid arthritis and so on), vitamin D deficiency, renal disease (history of kidney stones), cardiovascular disease, diabetes mellitus, and dementia [2].

Among workers, women employed in sedentary work constitute a priority target group for osteoporosis prevention. However, little attention has been paid to the prevention of osteoporosis. Research estimated that about 75% of osteoporosis cases are still undiagnosed or are diagnosed only when a bone fracture occurs [4]. For this reason, the detection of early signs of bone loss appears to be crucial to minimize serious health consequences.

The current gold standard to assess bone mass density (BMD) is dual-energy X-ray absorptiometry (DEXA). Screening for osteoporosis by DEXA has been shown to be a cost-effective approach in high-risk populations; however, it remains unclear whether DEXA is suitable for whole-population screening [5]. An alternative to DEXA is represented by Bindex, which is a new generation of pulse-echo ultrasound (PEUS) measuring the bone density at the proximal tibia level [6,7]. PEUS is less accurate than DEXA when measuring BMD. However, being less expensive than DEXA, PEUS use could widen the inclusion criteria [8]. This study was a part of a two-year screening program aimed at preventing osteoporosis in the workplace through lifestyle interventions [4,9]. As an intermediate project output, we pursued the following objectives: (1) to identify workers at risk of osteoporosis; (2) to identify workers with pathological BMD scores measured with PEUS; and (3) to evaluate the economic sustainability of this early screening program.

## 2. Materials and Methods

### 2.1. Study Design and Participants

We conducted a cross-sectional study including 1050 people working in 118 healthcare facilities in Italy. A self-administered online questionnaire was defined addressing all known osteoporosis risk factors [10]. We performed cognitive interviews to ensure that the respondents understood the items as intended, and we piloted a test on both a population sample of 20 subjects (supplemental materials Annex 1). To maximize compliance with the completion of the questionnaire, three reminders were sent to the study participants.

Subjects with one major risk factor or two minor risk factors for osteoporosis (supplemental materials Annex 2) were invited for PEUS bone density measurement at the proximal tibia level. Based on the BMD values, the subjects were classified as healthy subjects (BMD > 0.783 g/cm²) and pathological subjects with suspected osteopenia (BMD ≤ 0.783 g/cm² and > 0.719 g/cm²) or osteoporosis (BMD ≤ 0.719 g/cm²). Subjects with BMD ≤ 0.783 g/cm² were invited to a medical specialist visit that could include coaching and lifestyle interventions for workers with suspected osteopenia and drug prescriptions for workers with osteoporosis. A cost estimation was conducted by means of the full costing method. Full costing is an accounting method used to determine the complete end-to-end cost of producing products or services. The full cost analysis, in accordance with the estimate reported in the literature [7], included the time of the healthcare workers, the use of the Bindex tool, the organization time, and the questionnaire design.

### 2.2. Statistical Analysis

The collected variables were reported as absolute number (*N*) and percentage (%). To calculate the *p*-value, the odds ratio (OR) and the confidence level at 95% (CL95%) testing the association between the outcome variable BMD ≤ 0.783 g/cm^2^ and the risk factors investigated were performed using the univariate logistic regression model, the Chi-square, or the Fisher test, as appropriate. Statistical analysis was performed using STATA software (MP 13 version-StataCorp. 2013. Stata Statistical Software: Release 13. College Station, TX: StataCorp LP) in compliance with the Italian personal data privacy legislation.

### 2.3. Ethical Considerations

The ethical approval for the project was obtained from the Ethics Committee A.O.U. “Maggiore della Carità” (protocol code 584/CE).

## 3. Results

The responder rate was 60.4% (634/1050). The population features are shown in Table 1. According to the measured risk factors, 436 workers were invited to a PEUS scan and 300 agreed to participate. Based on the BMD scores, we identified 240 healthy individuals (80%, BMD > 0.783 g/cm²), and 60 with a significant bone loss: 47 people with osteopenia (15.7%, 0.783 ≤ BMD > 0.719 g/cm²) and 13 osteoporotic workers (4.3%, BMD ≤ 0.719 g/cm²). The features of the subjects with BMD ≤ 0.783 (60) and the risk factor distribution are shown in Table 2.

Regarding the statistical analysis, we observed a significant association between high risk workers with a ≥ 2 questionnaire score and a condition of reduced bone density with BMD ≤ 0.783 (OR = 1.83, 95%CL = 1.01–3.29, *p* = 0.043). As shown in Table 2, we observed a significant association between a lower BMD and the following variables: gender female, older ages, and menopause.

Cost estimation:

1. Using actual Italian data [11], the mean cost per one fracture in Italy is currently 16,785 euros; thus, we can estimate a theoretical cost of 100,710 euros in 20 years related to pathological fractures for six pathological subjects.
2. The early treatment of osteoporosis could avoid 24,170 euros of such costs (24% of 100,710 euros) [2,11].
3. The yearly cost of the screening program was 8242.00 euros (time of the healthcare workers, use of the Bindex tool, organization, and questionnaire design) [7];
4. The actual yearly cost of the treatments is 573.3 euros per person in Italy. We can estimate a theoretical cost of 3439.8 euros related to pathological fractures for six pathological subjects [2,11];
5. If we add this actual yearly cost of the treatments (3439.8) to the cost of our program (8242), we have a total of 11,681.8 euros;
6. If we compare the avoidable costs of fractures (24,170 euros) to this final amount (11,682 euros), we can measure a possible positive benefits/costs ratio of 2.07.

## 4. Discussion

As a major outcome, our study let us early identify 60 active healthcare workers with clinically significant bone loss who were not aware of their condition. This diagnostic anticipation constitutes an important element of value for the project, most of all for the workers, for the preventable negative outcomes caused by the fractures, and also for the employer and society in general, due to the related avoidable costs.

The analyses conducted in the study population revealed a significant association between the condition of bone fragility (BMD value ≤ 0.783 g/cm²) and the major risk factors for osteoporosis being gender female, age ≥ 50 years old, and menopause. Our screening program results are consistent with the evidence available in the literature regarding the populations at risk [4,12].

According to the literature [13], we can suggest that screening programs aimed for early diagnosis and treatment of osteoporosis could be remarkably cost-effective interventions. With the actual Italian fracture incidence rates in osteoporotic patients, we can reasonably expect that at least six fractures will occur in our sample in the next 20 years. If we consider that the mean cost per fracture in Italy is today 16,785 euros per person, we can estimate a theoretical cost of 100,710 euros related to pathological fractures for our sample (six people) [11]. According to the literature [2,11], we can also assume that the early treatment of osteoporosis could avoid approximately 24% of such costs (24,170 euros), and thus we can measure a possible positive benefit/costs ratio of 2.07.

However, our study has some limitations. First, we could not perform a sensitivity and specificity analysis of the questionnaire as the instrumental BMD assessment was not performed for the overall responder population. Even though we performed a pilot test for the questionnaire, submitting it to workers and health professionals, limitations related to the comprehensibility of the self-filled questionnaire could have persisted. The current evidence available on the use of the Bindex^®^ technology is limited [7]; therefore, the generalizability of our results could be partially biased. Last, in our study, the cost estimation was based on theoretical situations and estimated for only six patients.

## 5. Conclusions

In conclusion, based on our findings, we propose that PEUS-based screening performed in workplaces could be a possible cost-effective approach for the early identification of osteoporosis in the working population. Our findings also suggest that early identification of the at-risk population could both positively impact the quality of life of the person and the social network to which the person belongs, as well as limiting the indirect costs derived from osteoporotic fractures in the working population.

Further specific studies are needed for a better estimation of the possible benefits and costs of our screening program. However, we can say that the margin between the investment and results showed the advantage of the results, which indicates the benefits of this type of screening program.

### 5.1. What Do We Already Know about this Topic?

Osteoporosis is a major public health problem that is also expected to increase due to the aging population.

### 5.2. How Does Your Research Contribute to the Field?

Considering the ageing of the workforce, bone health promotion and osteoporosis prevention interventions in the workplace could be key elements of the public health responses to the osteoporosis’ burden. In particular, this study concerns the early screening of osteopenia and osteoporosis to find at-risk and sick subjects who otherwise would not be aware of their risk situation.

### 5.3. What Are the Implications of Your Research in Terms of Theory, Practice, or Policy?

Our research explores a bone health promotion intervention in the workplace by providing an initial estimation of early osteoporosis screening and subsequent interventions.

## Figures and Tables

**Table 1:** Total population features (*N* 634).

Population Features	*N* (%)
Female Male	519 (81.9) 115 (18.1)
Age: <40 40–49 50–59 ≥60 Mean for women Mean for men	183 (28.9) 223 (35.2) 178 (28.1) 50 (7.9) 46.3 43.6
BMI (kg/m^2^) <19 >19	83 (13.1) 551 (86.9)
Menopause: No Before 45 years old After 45 years old	337 (64.9) 40 (7.7) 142 (27.4)
Fragility fracture history	93 (14.7)
Familiarity fragility fracture	113 (17.8)
Smoker: No <10/die >10/die	431 (68) 108 (17) 95 (15)
Immobilization past 6 months: 15–30 days 31–60 days 61–120 days	9 (1.4) 5 (0.8) 2 (0.3) 2 (0.3)
Sedentary: extremely sedentary lifestyle occasional physical activity regular physical activity agonistic physical activity	39 (6.2) 251 (39.6) 331 (52.2) 13 (2.1)
Sun exposure: no no, but taking vitamin D yes	227 (35.8) 30 (4.7) 377 (59.5)
1 L/die water high calcium concentration intake (>200 mg/L)	440 (69.4)
Daily intake of food with high calcium content 0 portion 1–2 portions 3 or more portions	25 (3.4) 523 (82.5) 86 (13.6)
Alcohol use (wine/beer/spirits) 0 glasses 1–2 glasses More than 2	531 (83.8) 101 (15.9) 2 (0.3)
Daily food rich in salt	156 (24.6)
People declaring regular drug intake in the last 3 months	224 (35.3)
Number of diseases detected	175 (of which 1 organ transplantation)

**Table 2:** Total population features; associations between bone loss (0.783 g/cm^2^) and osteoporosis risk factors.

Variables (*N*, %)	Bindex Results ≤ 0.783, *N* (%)	*p*-Value *	Odds Ratio (95% CI)
Male (37, 12.3)	1 (1.7)	0.01	0.1 (0.0; 0.7)
Age class: <40 (60, 20) 40–49 (96, 32) 50–59 (113, 37.7) ≥60 (31, 10.3) Total (300, 100)	2 (3.3) 12 (20.0) 34 (56.) 12 (20.0) 60 (100)	1 0.1 <0.01 <0.01	1 4.1 (0.9; 19.2) 12.4 (2.9; 54.1) 18.3 (3.8; 89.3)
Menopause: No (133, 50.6) Yes, after 45 (103, 39.2) Yes, before 45 (27, 10.3) Total (263, 100)	13 (22.0) 38 (64.1) 8 (13.6) 59 (100)	<0.01 0.5 1	0.25 (0.1; 0.7) 1.39 (0.6; 3.5) 1
BMI, m²/kg > 19 (248, 82.7) Total (300, 100)	48 (80) 60 (100)	0.5	0.8 (0.4; 1.6)
Personal pathologic fracture history Yes (70, 23.3) Total (300, 100)	12 (20) 60 (100)	0.6	0.8 (0.4; 1.6)
Parental pathologic fracture history Yes (82, 27.3) Total (300, 100)	18 (30) 60 (100)	0.6	1.2 (0.6; 2.2)
Immobilization history:Yes (6, 2) Total (300, 100)	2 (3.3) 60 (100)	0.3	2.0 (0.4; 11.4)
Smoking: No (177, 59.0) Yes, <10/die (58, 19.3) Yes, >10/die (65, 21.7) Total (300, 100)	30 (50.0) 16 (26.7) 14 (23.3) 60 (100)	1 0.1 0.4	1 1.9 (0.9; 3.7) 1.3 (0.7; 2.7)
Physical activity: Sedentary (25, 8.3) Occasional (116, 38.7) Regular (155, 51.7) Agonistic (4, 1.3) Total (300, 100)	1 (1.7) 28 (46.7) 30 (50.0) 1 (1.7) 60 (100)	1 0.05 0.1 0.2	1 7.6 (1.0; 59.0) 5.8 (0.7; 44.3) 8 (0.4; 164.0)
Sun exposure: No (118, 39.3%) No, but taking vit-D (17, 5.7) Yes (165, 55) Total (300, 100)	18 (30.0) 4 (6.7) 38 (63.3) 60 (100)	1 0.4 0.1	1 1,7 (0.5; 5.8) 1.7 (0.9; 3.1)
Alcohol intake: 0 glasses (244, 81.3) 1–2 glasses (54, 18.0) More than 2 (2, 0.7) Total (300,100)	46 (76.7) 14 (23.3) 0 (0.0) 60 (100)	1 0.2 -	1 1.5 (0.75; 3.0)
Daily intake of food with high calcium content 0 portion (10, 3.33) 1–2 portions (248, 82.7) 3 or more portions (42, 42) Total (300, 100)	3 (5.0) 53 (88.3) 4 (6.7) 60 (100)	1 0.5 0.1	1 0.6 (0.2; 2.5) 0.1 (0.0; 1.3)

* Significant at *p* < 0.05.

## Data Availability

The data presented in this study are available on request to the authors. Some variables are restricted to preserve the anonymity of study participants.

## Supplementary Materials

The following are available online at https://www.mdpi.com/1660-4601/18/3/1368/s1.
