# Supplementary material for: The Early Detection of Osteoporosis in a Cohort of Healthcare Workers: Is There Room for a Screening Program?
Source: Int J Environ Res Public Health. 2021 Feb 2;18(3):1368. doi: 10.3390/ijerph18031368 (PMC7908569; doi:10.3390/ijerph18031368)
Supplement: Supplementary file 1 [file ijerph-18-01368-s001.pdf]

Table 1. Pilot study population features (N 20).

Population features.	N (%)
Total	20 (100)
Women	13 (65)
Man	7 (35)
Age	
< 40	5 (25.0)
40–49	7 (35.0)
50–59	5 (25.0)
≥ 60	3 (15.0)
Mean women	47.2
Mean men	44.8
BMI (kg/m2) < 19	5(25.0)
> 19	15 (75.0)
Menopause	
No	6 (46.2)
Before 45 years old	2 (15.4)
After 45 years old	5 (38.4)
Fragility fracture history	3 (15.0)
Familiarity fragility fracture	3(15.0)
Smoke No	12 (60.0)
<10/die	5 (25.0)
>10/die	3 (15.0)
Immobilization past 6 months	1 (5.0)
15–30 days	0 (0.0)
31–60 days	1 (5.0)
61–120 days	0 (0.0)
Sedentary: extremely sedentary lifestyle	4 (20.0)
occasional physical activity	5 (25.0)
regular physical activity	5 (25.0)
agonistic physical activity	1 (5.0)
Sun exposure: no	10 (50.0)
no, but taking vitamin D	8 (40.0)
yes	2 (10.0)
1l/die water high calcium concentration intake (>200mg/l)	11 (55.0)
Daily intake of food with high calcium content	
0 portion	3 (15.0)
1–2 portions	11 (55.0)
3 or more portions	7 (30.0)
Alcohol use (wine/beer/spirits)	
0 glasses	13 (65.0)
1—2 glasses	5 (25.0)
More than 2	2 (10.0)
Daily food rich in salt	4 (20.0)
People declaring regular drug intake in the last 3 months	7 (30.0)
Number of diseases detected	5

Table 2. Association analysis between survey-score and Bindex score (N 4).

.	Survey score, N. (%)	
	**≥1 and <2**	**≥2**
**Bindex score**		
**>0.783**	1 (50.0)	1 (50.0)
**≤0.783**	0 (00)	2 (100.0)
**Total**	1 (25.0)	3 (75.0)

Annex 2 – Criteria for inclusion in PEUS

	**Figure 1. major risk factor.<br>Age > 50 years + 1 or more minor risk factor<br>Age > 65 years**	**Male<br>Any age with 1 major risk factor<br>Age > 50 years + 2 or more minor risk factor<br>Age > 70 years**
**Major risk factor**	Menopause	
	Previous fragility fracture	Previous fragility fracture
	Family history of fractures	Family history of fractures
	Low body mass index <19	Low body mass index <19
	Smoking (> 10 cigarettes / day)	Smoking (> 10 cigarettes / day)
	Premature menopause <45 years	Premature menopause <45 years
	Immobilization protracted over time	Immobilization protracted over time
	Transplants	Transplants
	Diseases associated with osteoporosis	Diseases associated with osteoporosis
	Drugs associated with osteoporosis	Drugs associated with osteoporosis
**Minor risk factor**	Smoking (<10 cigarettes / day)	Smoking (<10 cigarettes / day)
	Alcohol (3 or more international units / day)	Alcohol (3 or more international units / day)
	Vitamin D deficiency	Vitamin D deficiency
	Sedentary lifestyle	Sedentary lifestyle
	Reduced calcium intake	Reduced calcium intake
	Excessive sodium intake	Excessive sodium intake
